# Unlocking Glioblastoma Secrets: Natural Killer Cell Therapy against Cancer Stem Cells

**DOI:** 10.3390/cancers15245836

**Published:** 2023-12-14

**Authors:** Yuanning Du, Karen E. Pollok, Jia Shen

**Affiliations:** 1 Medical Sciences Program, Indiana University School of Medicine, Bloomington, IN 47405, USA; yuandu@iu.edu; 2 Department of Medical and Molecular Genetics, Indiana University School of Medicine, Indianapolis, IN 46202, USA; kpollok@iu.edu; 3 Department of Pediatrics, Hematology/Oncology, Indiana University School of Medicine, Indianapolis, IN 46202, USA; 4 Department of Pharmacology and Toxicology, Indiana University School of Medicine, Indianapolis, IN 46202, USA; 5 Indiana University Melvin and Bren Simon Comprehensive Cancer Center, Indianapolis, IN 46202, USA

**Keywords:** GBM, GSCs, NK cells, immunotherapeutic strategy

## Abstract

**Simple Summary:**

Glioblastoma (GBM) is an aggressive brain tumor that is heterogenous at the cellular and molecular levels. It deftly invades the brain parenchyma making it challenging to treat. A cellular component of these tumors are specialized cells called glioblastoma stem cells (GSCs) that help the tumor grow and survive. Stopping these GSCs could make treating the tumor easier. Natural killer (NK) cells are like superheroes among immune cells; they can hunt down and destroy cancer cells. In lab tests, NK cells have shown promise in fighting GSCs, which could lead to better treatment for GBM. This review article will break down what GBM is, the problems in treating it, how GSCs fit in, and how NK cells might be the heroes we need to fight this tough tumor.

**Abstract:**

Glioblastoma (GBM) represents a paramount challenge as the most formidable primary brain tumor characterized by its rapid growth, aggressive invasiveness, and remarkable heterogeneity, collectively impeding effective therapeutic interventions. The cancer stem cells within GBM, GBM stem cells (GSCs), hold pivotal significance in fueling tumor advancement, therapeutic refractoriness, and relapse. Given their unique attributes encompassing self-renewal, multipotent differentiation potential, and intricate interplay with the tumor microenvironment, targeting GSCs emerges as a critical strategy for innovative GBM treatments. Natural killer (NK) cells, innate immune effectors recognized for their capacity to selectively detect and eliminate malignancies without the need for prior sensitization, offer substantial therapeutic potential. Harnessing the inherent capabilities of NK cells can not only directly engage tumor cells but also augment broader immune responses. Encouraging outcomes from clinical investigations underscore NK cells as a potentially effective modality for cancer therapy. Consequently, NK cell-based approaches hold promise for effectively targeting GSCs, thereby presenting an avenue to enhance treatment outcomes for GBM patients. This review outlines GBM’s intricate landscape, therapeutic challenges, GSC-related dynamics, and elucidates the potential of NK cell as an immunotherapeutic strategy directed towards GSCs.

## 1. Glioblastoma and Glioblastoma Stem Cells

Glioblastoma (GBM), widely recognized as one of the most aggressive primary brain tumors, has its origins in astrocytes, the supportive cells of the brain. This cancer type is characterized by a constellation of daunting features, including its remarkable capacity for rapid growth, invasive behavior, and tenacious resistance to conventional treatments [1,2,3]. Its impact on public health is profound, contributing to over 15,000 annual fatalities in the United States. The yearly incidence rate of GBM stands at an estimated seven cases per 100,000 individuals, with certain segments of the population witnessing an alarming rise in diagnoses [1]. The current gold standard for GBM treatment involves a combination of approaches, commencing with surgical resection, which aims to remove as much of the tumor as safely possible. Following surgery, adjuvant therapies are employed, including radiation therapy, which utilizes high-energy rays to target and destroy cancer cells, and temozolomide chemotherapy, a drug used to hinder the growth of cancer cells [2,3,4]. It is crucial to underscore that this therapeutic amalgamation, while exhibiting efficacy, is not without its drawbacks, as it can lead to severe grade 3 or 4 hematologic side effects [5]. Additionally, despite the aggressiveness of these treatment modalities, the prognosis for patients afflicted with GBM remains disheartening, with a median survival period hovering around 12 to 15 months following diagnosis. With a five-year survival rate of approximately 9.8% using the combination of temozolomide and radiotherapy, or 1.9% with radiotherapy alone, patients face distressing symptoms [1]. These include severe headaches, seizures, neurocognitive decline, and focal neurologic deficits [6]. The clinical relevance of studying GBM is underscored by its dismal prognosis and the paucity of effective treatment options. Therefore, it is paramount to intensify research efforts to ameliorate the prospects of patients locked in a relentless battle against this formidable disease.

**Figure 1:**
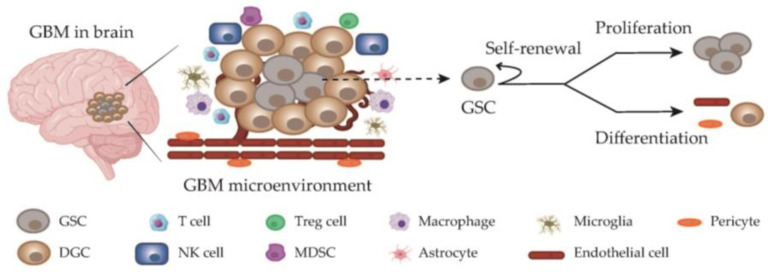
Glioblastoma microenvironment. The tumor cells, including GSCs and DGCs (differentiated glioblastoma cells), interact with various types of cells in the tumor microenvironment. GSCs exhibit the capacity for self-renewal, proliferation, and differentiation into diverse cell types within the glioblastoma microenvironment.

In the intricate landscape of GBM tumors, a unique subgroup known as GBM stem cells (GSCs) exists. These GSCs hold a pivotal role in shaping the cellular composition of GBM, and their distinct features set them apart within the tumor microenvironment. Much like conventional stem cells, GSCs possess an extraordinary capability for both self-renewal and differentiation into various cell types, including endothelial cells, pericytes, and other differentiated GBM cells (DGCs) (Figure 1), contributing significantly to the tumor’s inherent heterogeneity. Moreover, GSCs are distinguished by their exceptional proficiency in initiating and propagating tumors. This heightened tumorigenic potential places GSCs at the forefront of GBM’s development and progression, emphasizing their central role in the aggressive nature of this disease [7,8].

Researchers are actively investigating the origin of GSCs, and current understanding suggests that these cells may emerge from several potential sources [2]. First, GSCs can originate from normal neural stem cells, which are naturally present in the brain and have the capacity to generate various cell types, including astrocytes, oligodendrocytes, and neurons. Second, GSCs might also derive from more differentiated glial cells, which serve supportive functions in the central nervous system. The transformation of these normal neural or glial cells into GSCs involves an interplay of genetic mutations and epigenetic alterations. These genetic mutations and epigenetic modifications can lead to the reprogramming of the affected cells, endowing them with stem cell-like properties. These properties include self-renewal, enabling the cells to produce identical copies of themselves, and the potential to differentiate into diverse cell types, including endothelial cells, pericytes, and other DGCs, found within GBM tumors.

The pivotal role of GSCs in driving tumor growth, contributing to therapy resistance, and recurrence is widely acknowledged [9,10,11]. Targeting GSCs has become increasingly recognized as a potential strategy in the fight against GBM. Yet, this approach is not without its complexities. GSCs are a diverse group with inherent heterogeneity, employing various mechanisms to resist therapeutic interventions. Moreover, they engage in intricate interactions within the tumor microenvironment, making them particularly challenging as therapeutic targets.

GSCs display a remarkable diversity in both their outward characteristics and their functional properties [9,11]. Traditional methods have often relied on single-surface markers like CD15, CD44, CD133, and α6 integrin to isolate GSCs [9,12,13,14,15,16,17]. However, the limited specificity and sensitivity of these markers have proven insufficient to capture the full spectrum of GSC subpopulations [9,11,12]. Recent research has classified GSCs into distinct molecular subtypes, including proneural and mesenchymal. Notably, the mesenchymal subtype is associated with a more aggressive phenotype that contributes to disease progression [9,18,19,20]. Moreover, GSCs exhibit a high degree of epigenetic plasticity and the potential for interconversion between differentiated non-GSCs and GSCs. This highlights the dynamic nature of GSCs in shaping tumor heterogeneity [12]. Advances in single-cell analysis have deepened our understanding of GSC heterogeneity, revealing the presence of discrete subpopulations within the GSC landscape. Among these, proliferative GSCs (pGSCs) and quiescent GSCs (qGSCs) have been identified, each potentially playing distinct roles in tumor progression and recurrence [21,22,23]. Functional validation is a critical step in ensuring that enriched cells genuinely exhibit the functional characteristics of cancer stem cells. Various methods, both in vitro and in vivo, are employed to assess these cancer stem cell traits, which include self-renewal, proliferation, and the capacity to replicate the intricate nature of the original GBM tumor. The neurosphere formation assay is an in vitro test that can prove valuable for assessing GSC proliferation and self-renewal. Neurosphere formation serves as a reliable indicator of self-renewal capacity, particularly when performed with limiting dilution, enabling the determination of stem cell frequency. However, these assays may not comprehensively represent the GSC cellular hierarchy and lack the ability to replicate the complex GBM tumor microenvironment. The gold standard for pinpointing GSCs involves transplanting a limited number of cells into an orthotopic model, where they must be capable of regenerating the diverse cell types found in the original GBM patient’s tumor. This ability to recreate tumor heterogeneity is crucial because populations of transit-amplifying cells can form tumors but will only generate cells specific to their lineage.

The formidable resistance exhibited by GSCs to traditional therapies, such as radiation and chemotherapy, represents a significant hurdle in the clinical management of GBM [12,24,25,26]. These GSCs possess intrinsic resistance mechanisms that fortify their survival and uncontrolled growth. Notably, one key mechanism driving radioresistance in GSCs is the preferential activation of DNA damage checkpoint responses and an enhanced DNA repair capacity [7]. This is evidenced by the significantly increased phosphorylation of checkpoint proteins such as ataxia-telangiectasia-mutated (ATM), Rad17, Chk1, and Chk2 in GSCs compared to non-GSC cells within the tumor. Consequently, GSCs exhibit a more efficient DNA damage repair process compared to non-GSC cells, contributing to GSC survival. GSCs were observed to exhibit an upregulation of O6-methylguanine-DNA methyltransferase (MGMT) [27,28,29], a pivotal factor responsible for mending the primary cytotoxic damage caused by temozolomide [30,31,32,33], a commonly used chemotherapy drug in the treatment of GBM [2,3,4]. This heightened MGMT activity is often driven by the increased activation of the nuclear factor kappa-light-chain enhancer of activated B cells (NF-κB) [34], a transcription factor that plays a central role in regulating numerous cellular processes, including inflammation and proliferation [29,35,36]. Although NF-κB expression did not consistently vary in GSCs compared to differentiated GBM cells at the mRNA level, GSCs displayed increased levels of NF-κB subunit p65 protein [29]. Moreover, using the NF-κB inhibitor BAY 117082 or siRNA-mediated gene silencing could significantly reduce MGMT protein levels in GSCs [29]. Notably, increased MGMT expression in GSCs leads to resistance not just against temozolomide but also other therapies causing DNA damage [33,37]. Furthermore, GSCs exhibit a remarkable ability to undergo metabolic reprogramming, adapting their metabolic processes to sustain their aggressive phenotype and resist the effects of conventional therapies [38,39]. Previous studies established nicotinamide adenine dinucleotide (NAD^+^) metabolism alterations as a key factor in GSC radioresistance. GBM tumors and patient-derived GSCs notably exhibit high expression of nicotinamide phosphoribosyltransferase (NAMPT), a critical component in NAD+ synthesis. This increased NAMPT expression potentially leads to radioresistance [40]. Notably, resistant GSCs might transfer NAMPT to radiosensitive GBM cells via microvesicles (referred to as NAMPT-high MVs) [41], thereby contributing to radioresistance mechanisms. While metabolic strategies aimed at targeting GSCs have shown promise, they face the considerable challenge of overcoming the compensatory and adaptive responses mounted by these resilient cells [42,43]. The network of resistance mechanisms utilized by GSCs highlights the pressing demand for creative strategies to overcome their robust defenses and elevate the effectiveness of GBM treatment.

The tumor microenvironment of GBM consists of various cell types (Figure 1), each playing distinct roles in the progression and treatment resistance of the disease. These cells include GSCs, DGCs, T cells, NK cells, tumor-associated macrophages and microglia, endothelial cells, regulatory T cells (Tregs), myeloid-derived suppressor cells (MDSCs), and more [44,45]. Understanding the interplay between these diverse cell populations is crucial for developing effective treatment strategies for GBM [46]. GSCs tend to thrive within specialized protective microenvironments, with periarteriolar niches being particularly noteworthy in this regard, as they provide a nurturing haven that bolsters the survival of these cells while simultaneously enhancing their resistance to treatment regimens [47,48,49,50]. The intricate interplay between GSCs and their microenvironment essentially establishes a sanctuary for these cells, allowing them to evade the impacts of therapeutic interventions. In this intricate interaction between GSCs and their supportive niches, it becomes evident that more effective therapeutic strategies must extend beyond solely targeting the GSCs themselves; it is equally imperative to address the sheltering microenvironments that sustain these cells in order to make substantial progress in the treatment of GBM.

## 2. Natural Killer Cells in Cancer Immunotherapy

Natural killer (NK) cells are a unique subset of lymphocytes that play a crucial role in the immune system’s defense against infections and malignancies. NK cells originate from hematopoietic stem cells (HSCs) in the bone marrow. NK cell development involves a series of differentiation and maturation stages [47,51]. Specifically, the process begins with HSCs that can differentiate into common lymphoid progenitors (CLPs), which subsequently commit to the NK cell lineage. The differentiation process is orchestrated by a combination of transcription factors and cytokines, including interleukin-15 (IL-15). IL-15 is essential for NK cell development, survival, and activation. As NK cells mature, they acquire specific surface receptors, such as killer cell immunoglobulin-like receptors (KIRs) and natural cytotoxicity receptors (NCRs), which allow them to recognize a broad spectrum of target cells. These receptors are essential for the discrimination between healthy cells and those that may be infected or malignant. Once fully matured, NK cells are released into the bloodstream and widely distributed throughout various tissues, including the bone marrow, liver, spleen, and lymph nodes.

**Figure 2:**
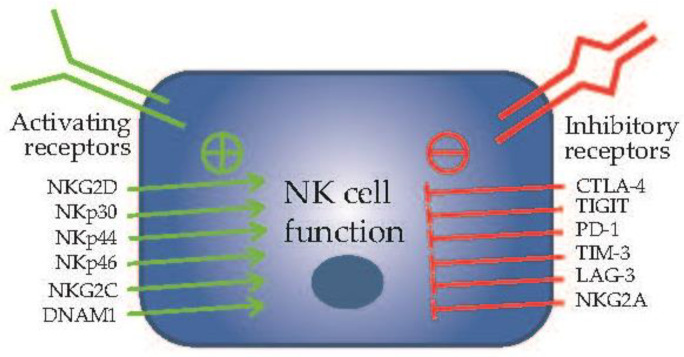
The activating receptors and inhibitory receptors related to NK cells regulatory signaling. The NK cell activating receptors include NKG2D, NKp30, NKp44, NKp46, etc. (**left**), and the receptors of inhibitory receptors include CTLA-4, TIGIT, PD-1, TIM-3, etc. (**right**).

NK cells serve as vigilant sentinels, continuously surveying their microenvironment for the presence of cells displaying abnormal surface markers or signs of distress. They are equipped to recognize stress-induced ligands found on the surface of target cells. NK cells operate based on signals received from two types of receptors, known as activating and inhibitory receptors [52]. Activating receptors include NKG2D, NKp30, NKp44, NKp46, NKG2C, DNAM1, and more, while inhibitory receptors consist of CTLA-4, TIGIT, PD-1, TIM-3, LAG-3, NKG2A, and CD96, among others [53] (Figure 2). Upon encountering a potential threat, NK cells employ their unique cytotoxic abilities to eliminate the target. They can directly induce target cell death through the release of cytotoxic granules containing perforin and granzymes, which trigger apoptosis in the affected cell. Additionally, NK cells can stimulate other immune cells to enhance the overall immune response. This includes interactions with dendritic cells, cytotoxic lymphocytes, and macrophages, enhancing the collective immune response against cancerous cells [54,55]. Therefore, NK cells hold a unique position, acting as crucial intermediaries that bridge the realms of both innate and adaptive immunity, exerting their influence on both fronts [51].

The dysregulation of NK cells has been implicated in the pathogenesis of various autoimmune diseases, such as ankylosing spondylitis, rheumatoid arthritis, and systemic lupus erythematosus [48]. Beyond autoimmunity, in the context of organ transplantation, NK cells, including the FcyRIII+ subset, assume a central role in immune responses, potentially influencing the acceptance or rejection of grafts [49]. Individuals with compromised NK cell activity or NK cell deficiencies have been identified as having a heightened risk of developing cancer [50,56,57]. Moreover, the involvement of NK cells in the regulation of metastasis has been extensively documented, suggesting their paramount importance in the context of tumor progression [58,59,60]. These multifaceted roles of NK cells underscore their significance in immune regulation, both in health and disease.

Immunotherapies have brought groundbreaking changes to cancer treatment, with T cell therapy and NK cell therapy emerging as prominent players. Due to their inherent capacity to recognize and eliminate tumor cells, NK cell-based therapies have distinct advantages: (1) T cell-based immunotherapies can lose their effectiveness when the specific antigen they target is absent. Prolonged treatment can lead to initial responses when the antigen is present, but the development of antigen shedding and the emergence of tumor escape variants eventually contribute to therapy resistance and tumor progression [61]. Unlike T cells, NK cells have the unique ability to act without prior activation or immunization, obviating the need for specific antigen recognition [54,59,62,63]. NK cells’ adaptability is particularly advantageous in combating heterogeneous tumors with varied antigen profiles, making NK cell therapy a versatile option for a wide spectrum of tumors. (2) Graft-versus-host disease (GvHD) is a condition where transplanted donor cells recognize the recipient’s body as foreign, potentially causing severe health problems. T cell-based immunotherapies can lead to GvHD if the transferred T cells, typically engineered for anti-cancer purposes, mistakenly attack healthy tissues, as they may lack perfect targeting selectivity. In contrast, NK cell therapy has an advantage in this regard. It often employs allogeneic NK cells from healthy donors, reducing the risk of GvHD. GvHD NK cell therapies exhibit greater specificity in targeting cancer cells while sparing healthy tissues, thus lowering the likelihood of GvHD and graft-related complications [52,62]. (3) T cell-based immunotherapies necessitate the extraction, modification, and expansion of patient-specific T cells, a process that can be time-consuming and challenging in the context of rapidly progressing cancers. In contrast, NK cells are renowned for their quick response to threats and their cytotoxic activity does not hinge on these time-consuming processes [52,62]. The swift response of NK cells can be a critical factor, especially in cases where urgent intervention is required. (4) Generally, NK cells have a shorter lifespan when compared to T cells. Unlike T cells, which can linger in the body for extended durations, NK cells tend to have a more limited lifespan. This quality is beneficial because it diminishes the risk of long-term side effects [52,62]. In situations where concerns about treatment-related complications arise, the shorter lifespan of NK cells can mitigate the dangers associated with sustained immune responses. These characteristics establish NK cell therapy as a contender in the battle against cancer and a valuable addition to the array of immunotherapeutic strategies available.

Various approaches have been investigated to boost NK cell performance and intensify their anti-tumor capabilities [64]. These strategies encompass methods like the ex vivo activation, expansion, and genetic modification of NK cells. Researchers have used cytokines and antibodies to stimulate NK cell function, successfully establishing uniform NK cell lines derived from both cancer patients and healthy donors. Another example is the successful engineering of cytokine-inducible SH2-containing protein (CIS) deletion in human primary NK cells using CRISPR/Cas9, which significantly enhances NK cell-mediated anti-tumor effects, particularly in GBM [65]. Among these techniques, chimeric antigen receptor (CAR)-NK cells stand out as a particularly promising genetically modified variant, showing a remarkable enhancement in their capacity to target and eliminate tumor cells. At the core of CAR-NK therapy is the concept of equipping NK cells with chimeric antigen receptors, thereby enhancing their tumor-targeting capabilities. The process of CAR-NK therapy commences with the extraction of NK cells from the patient’s own body or a compatible donor. These isolated NK cells are then genetically modified in the laboratory, where they are endowed with chimeric antigen receptors. These receptors are specifically designed to recognize unique antigens found on the surface of cancer cells, essentially acting as homing devices. The integration of chimeric antigen receptors equips the NK cells with the ability to recognize and bind to these cancer-specific antigens. Following the genetic modification, the CAR-NK cells are cultured and expanded to attain a sufficient quantity for therapeutic use. Once this robust population of CAR-NK cells is achieved, it is administered to the patient through infusion. After infusion, these engineered NK cells circulate throughout the patient’s body, actively seeking and targeting cancer cells. When a CAR-NK cell encounters a cancer cell that displays the corresponding antigen, it latches onto the cancer cell and unleashes a potent cytotoxic response to kill the cancer cell. The beauty of CAR-NK therapy lies in its precision and versatility. The CARs can be customized to target a range of specific antigens, allowing for tailored treatment approaches [64,65]. This approach shows promise in the treatment of various cancers, including challenging cases involving cancer stem cells, as the engineered NK cells can effectively track down and eliminate these elusive, treatment-resistant cells. These advancements in NK cell-based immunotherapies hold promise for more effective and adaptable approaches to cancer treatment.

The field of NK cell-based therapies has witnessed significant advancements and outcomes in various cancer types, as evidenced by results from clinical trials [66]. Harnessing the full potential of NK cells in cancer immunotherapy presents a paradigm-shifting approach to cancer treatment.

## 3. Natural Killer Cells Targeting of Glioblastoma Stem Cells

The potential of NK cells in cancer therapy has emerged as a groundbreaking frontier in medical research, with notable findings underscoring their unique role in targeting cancer stem cells in multiple solid tumors [67]. The activation of NK cells through the administration of IL-2 and IL-15 has unveiled their exceptional ability to detect and eliminate cancer stem cells [68]. This phenomenon has been observed across a spectrum of cancer types, including but not limited to breast cancer, colon cancer, and melanoma. Of particular significance is the investigation into the presence of NK cells within the GBM microenvironment. Research has revealed that specialized NK cells are strategically located in proximity to perivascular niches within patient-derived GBM tissue sections [69]. This revelation implies that these NK cells have the potential to overcome the formidable blood–brain barrier and reach the elusive GSCs situated within the brain. To unravel the intricate mechanisms governing the recognition and elimination of GSCs by NK cells, in-depth studies have delved into the structure and function of NK cell receptors. These receptors, which encompass both activating and inhibitory types, play a crucial role in the NK cells’ ability to identify and bind to GSCs. Moreover, studies have demonstrated that infiltrating NK cells can effectively target and lyse glioblastoma stem-like tumorspheres, thereby amplifying the overall effectiveness of chemotherapy [69]. Collaborative research efforts involving NK cells and cytotoxic drugs like cisplatin or temozolomide have highlighted that GSCs exposed to NK cell supernatants display heightened sensitivity to these chemotherapeutic agents. These findings illuminate the potential synergy between NK cells and standard cancer treatments, offering new avenues for improving patient outcomes in the battle against highly aggressive GBM [70,71,72,73].

Nevertheless, the effectiveness of NK cells in targeting and eliminating GSCs can be contingent upon the level of activation of these immune cells within the specific tumor microenvironment. This context-dependent responsiveness adds a layer of complexity to the overall efficacy of NK cell-based therapies [74]. First, GSCs have developed a range of sophisticated mechanisms to evade the vigilant surveillance of NK cells. Notably, they can create an immunosuppressive milieu within the tumor microenvironment, effectively shielding themselves from NK cell attacks. This immunosuppression is often orchestrated by the presence of MDSCs, a cell population known for its immune-inhibitory properties. MDSCs play a pivotal role in subduing the activity of NK cells, hampering their ability to target and eliminate GSCs. This immunosuppressive shield contributes to the resilience of GSCs within the tumor [55,75,76,77,78]. Furthermore, metabolic changes associated with tumor progression can impose additional challenges on NK cell-mediated eradication of GSCs. The altered metabolic landscape within the tumor microenvironment can inactivate NK cells but provide GSCs with a survival advantage by rendering them less susceptible to NK cell-induced cell death. The specific metabolic alterations that foster GSC survival are an active area of research and are being explored as potential targets to enhance the efficacy of NK cell therapy. Therefore, in the quest to maximize the killing activity of NK cells against GSCs, strategies are being developed to bolster their infiltration into the tumor site and to counteract the immunosuppressive signals propagated by GSCs and the tumor microenvironment [58]. For instance, targeting the αv integrin/TGF-β axis shows promise in enhancing the function and immune responses of adoptively transferred NK cells against GSCs [79]. Genetically engineered NK cells, such as CAR-NK cells, are gaining attention for their potential to target GSCs and GBM [64,80,81]. Recent studies have ventured into exploring the combination of NK cell-based therapies with other therapies. These include the integration of NK cell therapies with autophagy inhibitors [82] and antibodies [83], which have displayed the potential for synergistic effects in enhancing the overall anti-tumor immune response.

## 4. Conclusions and Future Perspectives

GBM is an exceedingly aggressive and malignant brain tumor that has posed a significant challenge in the field of oncology. One of its most formidable adversaries within this relentless tumor is a subpopulation of cells known as GSCs. These GSCs possess unique characteristics, including self-renewal and tumorigenic properties, making them a driving force behind GBM’s relentless growth and frequent recurrences. NK cells are a vital component of the body’s immune system, serving as a frontline defense against infections and cancer. These innate immune cells are renowned for their ability to swiftly recognize and eliminate abnormal or infected cells without prior sensitization. Given their unique properties, NK cells have become a focal point in the development of innovative immunotherapies for cancer. Since NK cells can kill the cancer stem cells from various tumor types, NK cell-based therapies offer hope for more effective and targeted approaches to eradicate GSCs, ultimately translating into better prospects for individuals battling GBM.

**Figure 3:**
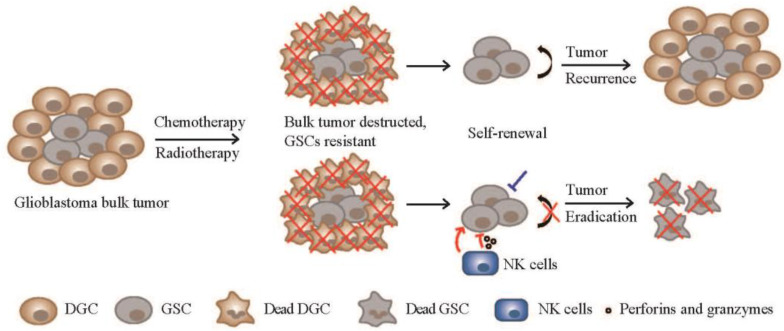
A “one-two punch” strategy to target GSCs and glioblastoma recurrence. Conventional therapies for glioblastoma, such as chemotherapy and radiotherapy, can eliminate most of the DGCs in the tumor mass. However, GSCs may survive and undergo self-renewal, leading to tumor relapse. The “one-two punch” strategy aims to block the GSC self-renewal and simultaneously activate NK cells to eliminate GSCs and glioblastoma.

While the prospect of NK cell targeting of GSCs holds promise, the development of efficient NK cell-based therapies targeting GSCs faces challenges related to GSCs themselves, NK cells, and the complex microenvironment within GBM tumors. In the future, these research directions have the potential to advance the development of more effective NK cell-mediated therapies aimed at targeting GSCs and GBM: (1) Delving deeper into NKG2D’s function. NKG2D is a potent NK activating receptor on NK cells. When NKG2D interacts with its ligands on target cells, it activates the NK cell, leading to the release of cytotoxic granules and the destruction of the target cell. Further research into the detailed activation mechanisms of NKG2D as well as exploring how to harness NKG2D and its ligands in concert with other therapies can offer insights into optimizing this process for enhanced GSC killing. (2) Optimizing NK cell sources. Selecting the right source for therapeutic NK cells in adoptive transfer is a critical factor. Allogeneic NK cells obtained from healthy donors offer an option due to their safety record and decreased potential for GvHD [84]. These cells could potentially serve as convenient “off-the-shelf” resources for NK cell-based therapies. It is imperative to continue refining and streamlining this sourcing process. (3) Genetic engineering. Genetic engineering of NK cells, particularly the development of CAR-NK cells, holds significant potential. These engineered cells can be tailored to specifically target GSCs, thereby enhancing their anti-tumor activity. CAR-NK cells present advantages such as lower GvHD risk and reduced off-target toxicities, making them an exciting avenue for further exploration [85]. (4) Combination therapies. The synergy between NK cell-based therapies and other immunotherapies or targeted therapeutic strategies is a field ripe for exploration. For instance, combining NK cell therapies with approaches that counteract the immunosuppressive tumor microenvironment or address the unique functionalities of GSCs can lead to improved treatment outcomes. In our lab, we have been pioneering a “one-two punch” strategy that not only inhibits GSC self-renewal but also enhances the attraction and killing function of NK cells against GSCs (Figure 3). This approach offers a rational basis for the development of combined targeted therapy and immunotherapy for GBM patients.

Exploring these prospective research avenues, with an emphasis on enhancing NK cell activation, genetic engineering, and the utilization of combination therapies, is of paramount importance in advancing the development of potent NK cell-based immunotherapies for combating GSCs. These avenues hold great promise in moving us closer to achieving enhanced patient outcomes in the challenging battle against formidable GBM.
